# Incidence of Slipped Capital Femoral Epiphysis in Crete (2021–2024): A Prospective Population-Based Study with a Focused Review of the Recent Literature

**DOI:** 10.3390/children13030362

**Published:** 2026-03-03

**Authors:** Emmanouela Dionysia Laskaratou, Charalampos Konstantoulakis, Ioannis Sperelakis, Georgios M. Kontakis, Nikolaos Tzanakis, Rozalia Dimitriou

**Affiliations:** 1 Department of Orthopaedics and Traumatology, Medical School, University of Crete, 70013 Heraklion, Greece; ysperelakis@uoc.gr (I.S.); kontak@med.uoc.gr (G.M.K.); 2 Orthokinesis Private Practice, 73133 Chania, Greece; bekonst@yahoo.com; 3 Department of Respiratory Medicine, University Hospital of Heraklion, 71500 Heraklion, Greece; tzanakis@med.uoc.gr

**Keywords:** slipped capital femoral epiphysis, incidence, epidemiology, population-based study, Crete, Greece, adolescence

## Abstract

**Background:** Slipped capital femoral epiphysis (SCFE) is an adolescent hip disorder of high clinical importance in which diagnostic delay may lead to irreversible complications and long-term morbidity, and its incidence varies considerably across populations. **Objective:** The primary aim of this study was to provide contemporary, population-based incidence estimates of SCFE among children aged 6–16 years in Crete (2021–2024), addressing the lack of data from Greece and Southern Europe. A secondary aim was to contextualize these findings through a focused synthesis of international incidence reports published since 2015. **Methods:** All pediatric orthopedic specialists in Crete reported incident SCFE cases, all of which were radiographically confirmed. All confirmed cases of SCFE were documented. Annual population data for children aged 6–16 years were obtained from the Hellenic Statistical Authority. Incidence rates were expressed per 100,000 children aged 6–16 (95% CI, exact Poisson). A structured literature search (2015–May 2025) identified recent studies reporting SCFE incidence. **Results:** Eleven children with SCFE were identified (14 affected hips; three bilateral cases), corresponding to an incidence of 4.6 per 100,000 person-years (95% CI 2.5–7.8) among children aged 6–16 years. Mean age at presentation was 10.72 years, 64% were female, and 82% were obese, with comorbidity findings recorded. The literature review (2015–May 2025; 14 studies) demonstrated substantial geographic variation in SCFE incidence, ranging from ~5 per 100,000 in Mediterranean settings to 57 per 100,000 boys aged 9–15 years in Sweden. The Cretan incidence rate was broadly consistent with other Mediterranean reports. **Conclusions:** SCFE is an uncommon condition in Crete, with incidence rates comparable to those of other Mediterranean populations. The geographically defined setting and relatively stable demographics support case ascertainment and the interpretability of the incidence estimates. Larger nationwide studies are warranted to better characterize SCFE epidemiology in Greece.

## 1. Introduction

Slipped capital femoral epiphysis (SCFE) is a clinically important adolescent hip disorder and remains a significant cause of long-term morbidity when diagnosis is delayed or treatment is suboptimal [1,2]. It is characterized by external and anterior displacement of the proximal femoral metaphysis relative to the capital femoral epiphysis through the growth plate, typically occurring during periods of accelerated growth [1,3]. The underlying mechanism of SCFE has not been fully elucidated, but its etiology appears to be multifactorial. During adolescence, several factors converge to render the physis particularly susceptible to slippage, including its more vertical orientation, which predisposes it to shear forces [4,5]. Obesity is the most consistently identified risk factor, increasing shear forces across a biomechanically vulnerable physis [6]. Endocrine and metabolic disorders, including hypothyroidism and pituitary abnormalities, further compromise physeal integrity and are overrepresented among affected children, particularly in those presenting outside the typical age range [6].

SCFE is commonly classified using three main criteria: the patient’s ability to bear weight, regardless of crutch use; the duration of symptoms; and the severity of the slip. SCFE is classified as stable when the patient is able to ambulate, with or without the assistance of crutches, and unstable when the patient is unable to bear weight, even with crutches [7]. Assessing the stability of SCFE is clinically important, as unstable SCFE is associated with a poorer prognosis and a significantly higher risk of complications [8]. Slip severity is graded, according to the percentage of femoral head displacement relative to the femoral neck, as mild (0–33%), moderate (34–50%), or severe (>50%). However, this method has significant inter-/intra-observer variability and is also influenced by patient position [9]. Based on symptom duration, SCFE is categorized as acute when symptoms have been present for less than three weeks, chronic when they have persisted for three weeks or more, and acute-on-chronic when there is a sudden aggravation of longstanding symptoms [10].

The incidence of SCFE varies significantly worldwide, ranging from as low as 5.0 per 100,000 in Turkey to sex-specific rates exceeding 40 per 100,000 in Northern Europe (44 per 100,000 girls and 57 per 100,000 boys aged 9–15 years in Sweden) [11,12]. SCFE most commonly presents between 10 and 16 years of age, concurrently with peak height velocity, and often manifests with non-specific symptoms such as hip, thigh, or knee pain and limping, or even prodromal symptoms prior to presentation [5,13,14]. While a male predominance has traditionally been reported, recent studies suggest that sex distribution may vary across populations. The average age at presentation is 12 years for girls and 13.5 years for boys [5,15]. SCFE is bilateral in 20% to 80% of cases reported worldwide [5].

Although SCFE has been extensively studied in North America and Northern Europe, contemporary population-based incidence estimates from Southern Europe—and Greece in particular—remain limited, which constrains the generalizability of existing figures to Mediterranean populations. Accordingly, the primary aim of this study was to estimate the incidence of SCFE among children aged 6–16 years in Crete during 2021–2024, with a secondary aim of contextualizing these findings through a focused review of the recent international literature.

## 2. Materials and Methods

### 2.1. Study in the Cretan Population

A population-based prospective incidence surveillance study was conducted in the region of Crete to determine the annual and overall incidence of SCFE during 2021–2024. Incident SCFE cases were ascertained prospectively throughout the study period through the island’s centralized pediatric orthopedic care pathway. Incidence was calculated using annual population denominators provided by the Hellenic Statistical Authority for each calendar year (2021–2024). The total population of Crete during the study period was approximately 624,400 on average. The annual denominators (population at risk), i.e., the population aged 6–16 years, were 77,264 in 2021, 75,489 in 2022, 75,109 in 2023, and 74,436 in 2024.

In Crete, any suspected case of SCFE is initially assessed by orthopedic surgeons with a specialization or specific interest in pediatric orthopedics. Definitive diagnosis was established by a panel of three pediatric orthopedic specialists based in the two largest regional units of the island (Heraklion and Chania), using standard clinical and radiographic criteria. These two public hospitals are the only centers in Crete where surgical treatment for SCFE is performed. Consequently, all confirmed cases requiring operative management are referred to these institutions, allowing for comprehensive case ascertainment within the defined population.

Incidence rates were calculated by dividing the number of newly diagnosed SCFE cases by the population at risk and were expressed as cases per 100,000 children. Ninety-five percent confidence intervals (95% CIs) for incidence rates were calculated assuming a Poisson distribution of rare events. Exact Poisson limits were derived using the chi-square method according to the following formulas [16]:

λL = (1/2T) χ^2^_0.025_,2k

λU = (1/2T) χ^2^_0.975_,2(k + 1)

where χ^2^ denotes the chi-square distribution; λL and λU are the lower and upper confidence limits of the Poisson mean, multiplied by 100,000; k represents the number of observed cases; and T is the population at risk. The corresponding incidence rate confidence limits were obtained by dividing λL and λU by the population at risk and multiplying by 100,000.

This design provides repeated annual incidence estimates at the population level; this was not intended as a longitudinal outcome study with follow-up beyond the index diagnosis and treatment.

#### 2.1.1. Unit of Analysis

Incidence was calculated at the patient level using incident SCFE cases. Patients who developed bilateral disease during the study period were counted once in the incidence numerator, while laterality (left/right) and bilaterality were recorded and reported descriptively. All patients diagnosed with SCFE during the study period were referred to one of the two pediatric orthopedic centers on the island and underwent operative management; accordingly, no conservatively managed (non-operative) SCFE cases occurred. Given that SCFE surgical care on the island is centralized in two public hospitals, this pathway supports comprehensive case ascertainment within the defined catchment area, although a small degree of under-ascertainment (e.g., care sought outside Crete or very mild/atypical presentations not reaching specialist assessment) cannot be entirely excluded.

#### 2.1.2. Ethics Approval and Consent

The study protocol was approved by the Bioethics and Ethics Committee of Heraklion University Hospital (approval No. 16/07-07-2021). Given the observational and population-based nature of the study, the requirement for informed consent was waived.

Participant privacy and confidentiality were maintained throughout the study period. Access to the study file was restricted to the study team and stored on password-protected institutional systems. No directly identifiable personal information was included in the study database or in any reports.

### 2.2. Review of the Literature

A focused narrative review of the literature was conducted to identify peer-reviewed studies reporting the incidence of SCFE. The databases searched were PubMed/MEDLINE and the Cochrane Library, covering the period from 1 January 2015 to 31 May 2025. The final search was performed in June 2025. No restrictions on study design were applied at the search stage.

PubMed/MEDLINE (full search string):

((“Slipped Capital Femoral Epiphysis”[Title/Abstract] OR “Slipped Upper Femoral Epiphysis”[Title/Abstract] OR SCFE[Title/Abstract] OR SUFE[Title/Abstract]) AND (“Incidence”[Title/Abstract] OR incidence[Title/Abstract] OR epidemiology[Subheading] OR epidemiolog*[Title/Abstract])) AND “2015/01/01”[Date–Publication]: “2025/06/30”[Date–Publication].

Cochrane Library (full search string):

((“Slipped Capital Femoral Epiphysis” OR “Slipped Upper Femoral Epiphysis” OR SCFE OR SUFE) AND (incidence OR epidemiolog*)) with publication year limits 2015–2025.

Titles/abstracts were screened, followed by full-text assessment against predefined eligibility criteria. Studies were eligible if they reported population-based incidence data for SCFE in pediatric or adolescent populations. Studies focusing primarily on surgical techniques, therapeutic outcomes, or conference abstracts were excluded. Reference lists of included articles were also screened to identify additional eligible studies. In total, 14 studies were identified.

Because this was a focused narrative review rather than a systematic review, we did not perform a formal risk-of-bias assessment or exhaustive database searching; therefore, relevant incidence studies may have been missed, and the synthesis should be interpreted as contextual rather than comprehensive. Accordingly, a PRISMA flow diagram was not produced.

## 3. Results

### 3.1. Incidence of SCFE in Crete (2021–2024)

During the study period (2021–2024), a total of 11 children were diagnosed with SCFE in Crete, accounting for 14 affected hips, including three cases of bilateral involvement. In 2021, the population of children aged 6–16 years in Crete comprised 77,264 individuals, among whom three new cases of SCFE were identified, corresponding to an annual incidence of 3.9 per 100,000 children (95% CI: 0.8–11.3). In 2022, three cases were recorded among 75,489 children, yielding an incidence of 4.0 per 100,000 children (95% CI: 0.8–11.2). In 2023, four cases were identified in a population of 75,109 children, resulting in an annual incidence of 5.3 per 100,000 children (95% CI: 1.5–13.8). Similarly, in 2024, four cases were diagnosed among 74,436 children, corresponding to an incidence of 5.4 per 100,000 children (95% CI: 1.5–13.8). Across the four-year study period (2021–2024), the overall incidence of SCFE in Crete was 4.6 per 100,000 children aged 6–16 years (95% CI: 2.5–7.8 per 100,000).

The mean age of SCFE presentation was 10.7 years (range 6–14 years old), while most of the individuals [7] were females (64%). Thyroid dysfunction was reported in four out of 11 (36%) patients, while three (27%) had at least one related comorbidity, such as early puberty [2] and panhypopituitarism due to a pituitary tumor in one individual. Ten of the eleven patients (91%) were classified above the 90th percentile for body weight according to age- and sex-specific reference standards. Vitamin D status was not systematically evaluated across the cohort (it was documented in only one case), precluding any meaningful interpretation of its potential association with SCFE in this population-based series (Table 1).

### 3.2. Review of the Literature

Across the included studies, the reported incidence of SCFE demonstrated substantial geographic variation. The lowest incidence to date was reported in Turkey by Tasci et al., with an estimated rate of 0.005% (equivalent to five cases per 100,000), based on cases diagnosed in children younger than 16 years between 2016 and 2023 [11]. In contrast, the highest sex-specific incidence was documented by Herngren et al. (2017) who reported a rate of 57.0 per 100,000 boys in the age group 9–15 [12]. In an earlier comprehensive review, Loder and Skopelja reported a peak incidence of 17.15 per 100,000 in the United States [17]. In addition, higher incidences have consistently been reported in Pacific Islander populations, including in American Samoa [18]. Differences in case definitions, age ranges, and national healthcare registries likely contribute to the wide variability in reported incidence rates.

The mean age at presentation reported across the included studies was 12.2 years. All studies consistently noted a younger age at diagnosis in female patients than in males (Table 2).

Obesity was identified as a major associated risk factor in the majority of the reviewed studies. Specifically, nine of the 14 studies reported either a high prevalence of obesity among patients with SCFE or an increasing incidence of SCFE parallel to rising obesity rates in the pediatric population [11,12,14,18,23,24,25,26,29]. Conversely, two studies reported a decreasing trend in the prevalence of obesity within the age groups included in the study [20,21].

Reporting of endocrinopathies, particularly hypothyroidism, was inconsistent across studies in terms of case definitions, the spectrum of conditions evaluated, and the level of detail provided. Kim et al. found that 4% of patients had hypothyroidism, and an additional 11.44% had other endocrinopathies such as parathyroid or pituitary gland disorders [22]. In contrast, Perry et al. reported a notably higher prevalence, with 27% of SCFE patients presenting with hypothyroidism [26]. Taşcı et al. emphasized the broader role of endocrine and metabolic abnormalities, reporting vitamin D deficiency in 69% of cases and other endocrinopathies in 11% of patients [11]. Collectively, these findings suggest substantial heterogeneity in how endocrine comorbidities were assessed and reported, which limits direct comparison of prevalence estimates across cohorts.

### 3.3. Symptom Duration, Stability, and Slip Severity in SCFE

Most of the included studies did not report detailed data on symptom duration at presentation. An exception was the study by Beharry et al., which reported a mean symptom duration of 196 ± 220 days, consistent with chronic SCFE [25]. Fedorak et al. reported a distribution of 45.5% acute and 54.5% chronic SCFE cases [18].

Across the reviewed studies, unstable SCFE was consistently less frequent than stable SCFE. Reported proportions of unstable SCFE ranged from 5.5% to 21% across the available cohorts [18,23]. Only three studies reported a classification of SCFE severity [12,18,23]. These studies consistently indicated that most SCFE presentations fall into the mild category, with moderate and severe slips occurring less frequently. Unstable slips accounted for approximately 6–21% of cases, depending on the cohort.

## 4. Discussion

This study provides the first prospective, population-based incidence estimate of slipped capital femoral epiphysis (SCFE) in Greece. Using prospective case ascertainment in Crete over a four-year period (2021–2024), we estimated incidence and described the clinical and demographic characteristics of incident SCFE cases and then interpreted these findings in the context of contemporary international incidence reports. Although Crete is an island, its population is ethnically and socioeconomically comparable to mainland Greece, with no known genetic distinctions relevant to the outcomes studied; nonetheless, some limitation in external generalizability cannot be fully excluded.

Our results indicate that SCFE remains an uncommon condition in this Mediterranean population, with an overall incidence of 4.6 cases per 100,000 children aged 6–16 years. These findings are consistent with data reported from other Mediterranean countries, such as Turkey (5/100,000) and Italy (2.9/100,000), reinforcing the observation that SCFE may be less prevalent in southern European populations compared to those in North America or northern Europe [11,27,29]. Crete constitutes a geographically defined catchment area with relatively stable demographics and centralized pediatric orthopedic surgical care, which supports case ascertainment for incidence estimation. Nevertheless, a small degree of under-ascertainment cannot be entirely excluded (e.g., patients seeking care outside the island or very mild/atypical presentations not reaching specialist assessment).

The at-risk population was defined as children aged 6–16 years, reflecting the age range during which SCFE most commonly occurs and preceding proximal femoral physeal closure [12]. This approach made it possible to capture the population at highest risk while minimizing the inclusion of age groups in which SCFE is rare and more frequently associated with atypical etiologies, such as endocrine disorders.

The mean age at presentation of SCFE in the study was 10.72 years, slightly younger than the mean age of 11–13 years commonly reported in international studies. Despite this slight difference, our data remained broadly consistent with the established epidemiology of SCFE. Females have been reported to present with SCFE at a younger age than males in several cohorts; therefore, the relatively young mean age in our series may have contributed to the observed female predominance. With respect to sex distribution, our cohort demonstrated a female predominance (64%), giving a female-to-male ratio of 1.8/1. This does not align with the male predominance reported in most large population-based studies, where male-to-female ratios of approximately 1.6:1 are typically observed [12,17]. This discrepancy should be interpreted cautiously and is most likely attributable to the small sample size rather than reflecting true population-specific characteristics of the population of Crete.

Obesity was present in 82% of patients in our series, highlighting its role as a major risk factor for SCFE. This observation is in accordance with the literature emphasizing obesity as a major risk factor for SCFE. Studies such as those by Herngren et al. (2017) and Navarre (2020) report similarly high obesity rates among SCFE patients [12,24]. In contrast, recent reports have described a stabilization or decline in obesity prevalence in certain populations [20,21]. Endocrine disorders were identified in a relatively high proportion of patients in this study, with thyroid dysfunction presented in 36% (4/11) and panhypopituitarism 9% (1/11). These proportions are substantially higher than those reported in large-scale studies. For example, Kim et al. (2023) reported hypothyroidism in 4% of cases and other endocrinopathies in 11.4%, while Tasci et al. (2024) noted non-thyroid endocrinopathies in 11% [11,22]. Vitamin D deficiency was reported in only one of the included incidence cohorts (69% in Taşcı et al.) [11]. Given that this factor was not consistently assessed or reported across studies, the scope for direct comparison or inference is limited. In our cohort, vitamin D testing was not performed systematically, and the available data were too sparse to permit meaningful interpretation. Multiple factors may contribute to this observed discrepancy, including the limited sample size, the inclusion of pediatric patients with complex comorbid conditions, and variability in diagnostic and screening protocols. Accordingly, these results should be regarded as descriptive and not interpreted as evidence of a definitive association at the population level. Regarding clinical presentation, all patients in our series presented with limping and hip pain, and 20% reported knee pain. Unstable SCFE accounted for 36% (4/11) of cases in the study, which is slightly higher than the rates reported by Perry et al. (21%) and Herngren et al. (15.83%) [12,23]. Similarly, the distribution of temporal classification demonstrated a predominance of acute presentations, with smaller proportions of acute-on-chronic and chronic cases, broadly aligning with previously published data [18]. As with other findings, these proportions are likely influenced by the limited number of cases in this study.

Bilaterality was less frequently observed in our series, with most cases presenting unilaterally and a predominance of right-sided involvement. This contrasts with reports from some cohorts in which bilaterality rates demonstrated in higher proportions, such as the previously referred to studies by Navarre and Beharry et al., with 45% and 68.5%, respectively [24,25]. Given the small cohort size, laterality findings should be interpreted descriptively rather than inferentially. Published cohorts have not reported a consistent side predominance, and laterality patterns appear variable between populations; therefore, the right-sided predominance observed in our series should be interpreted descriptively.

### Strengths and Limitations

The principal strengths of this study include its prospective, population-based design and the use of a geographically defined and demographically stable population, which likely enhanced case ascertainment and the accuracy of incidence estimates. The centralization of pediatric orthopedic care on the island and the multiyear study period further support the reliability of the findings. The study’s main limitation is the small number of cases, which restricts statistical precision and limits subgroup analyses. Although comprehensive collaboration across pediatric orthopedic services was achieved, the possibility of missed mild or atypical cases cannot be excluded. Although centralized pediatric orthopedic care supports comprehensive ascertainment, a small number of mild or atypical presentations may have been missed. These could include early or ‘pre-slip’ cases with subtle radiographic changes; patients presenting primarily with referred knee or distal thigh pain who were not promptly evaluated with hip imaging; and minimally symptomatic stable slips managed initially outside specialist pathways before referral. In addition, rare cases with atypical age or comorbidity profiles (e.g., endocrine/metabolic disorders) might be evaluated in non-orthopedic settings before orthopedic assessment. If such presentations did not reach the pediatric orthopedic centers during the ascertainment window, the incidence estimates would be biased toward underestimation; however, given the centralized referral and operative management pathway on the island, any under-ascertainment is expected to be limited. In addition, the absence of a control group precludes causal inference regarding associations between SCFE and identified risk factors. Nevertheless, even small population-based studies provide valuable epidemiological benchmarks for rare pediatric conditions.

## 5. Conclusions

In this prospective, population-based study, we provide the first incidence estimate of SCFE in Greece and describe the clinical and demographic profile of incident cases in Crete (2021–2024). Our findings contribute region-specific epidemiological data from Southern Europe and enable comparison with contemporary international incidence reports. Although the sample size was small, the observed distribution of patient characteristics and comorbidities is consistent with a multifactorial framework for SCFE. These data may inform regional service planning and support future multicenter surveillance and collaborative studies to refine incidence estimates and better characterize risk profiles in Mediterranean populations.

## Figures and Tables

**Table 1:** Clinical characteristics and associated risk factors of individual SCFE cases in Crete (2021–2024).

Individual	Gender (F/M)	Presentation Age (Years)	Weight (Percentile)	Thyroid Dysfunction	Other Health Problems	Athletic Activities	Limping	Hip Pain	Knee Pain	Temporal Classification	Stability	Severity	Affected Limb
1	F	9	>97th	No	n/a	n/a	Yes	Yes	n/a	Acute	Stable	Mild	Right
2	F	8	>97th	No	n/a	Yes	Yes	n/a	n/a	Acute	Stable	Mild	Both
3	F	6	>97th	No	n/a	n/a	Yes	Yes	Yes	Acute on chronic	Stable	Mild	Both
4	M	12	>95th	Yes	n/a	Yes	Yes	Yes	n/a	Acute on chronic	Unstable	Mild	Left
5	F	11	95th	No	n/a	Yes	Yes	Yes	n/a	Acute	Unstable	Mild	Right
6	M	14	>95th	No	n/a	n/a	Yes	Yes	n/a	Chronic	Stable	Mild	Right
7	F	12	>97th	Yes	n/a	Yes	Yes	Yes	n/a	Acute	Stable	Mild	Right
8	F	9	>97th	Yes	Early puberty	Yes	Yes	Yes	n/a	Acute	Unstable	Mild	Right
9	F	11	50th	No	Early puberty	n/a	Yes	Yes	n/a	Acute	Stable	Moderate	Both
10	M	14	90th	Yes	Panhypopituitarism	n/a	Yes	Yes	Yes	Chronic	Stable	Mild	Left
11	M	12	>97th	No	n/a	n/a	Yes	Yes	n/a	Acute	Unstable	Moderate	Left

F—female; M—male; NR—not reported; NA—not applicable.

**Table 2:** Incidence and mean age of presentation for slipped capital femoral epiphysis (SCFE) across different studies, by region and age group.

Reference	Period	Country/Region	Age (Years)	Incidence Number of Cases/100,000 Children	Mean Age of Presentation (Years) (Range): Males/Females
Taşci et al., 2024 [11]	2016–2022	Turkey	0–16	5	12.9 (7–16)/ 13.3:11.2
Fedorak et al., 2018 [18]	2005–2014	Hawaii/USA	5–14	5–14 years: 53.1 5–19 years: 33.6	12.3 (7.3–18.1)
Hwang et al., 2024 [19]	2002–2019	South Korea	0–14	Higher in children with endocrinopathy than without (37.1 versus 9.0)	11.6 ± 1.7 (5–16)/ 11.9:10.9
Singh et al., 2024 [20]	2011–2020	USA	<18	Increased from 24.6 to 59.6 between 2011 and 2020 *	11.93
Ravinsky et al., 2019 [21]	2002–2011	Canada (Ontario)	9–16	5.68	n/a
Kim et al., 2023 [22]	2009–2019	South Korea	9–14	Average incidence: 6.0 Boys: 8.4 Girls: 3.5	11.1 ± 1.8/ 11.3 ± 1.9:10.6 ± 1.5
Perry et al., 2022 [23]	06/2016–08/2017	Great Britain	6–18	Annual incidence: In England 3.44 In Wales 1.23 In Scotland 3.53	12.7 (11.4 to 13.8)
Navarre, 2020 [24]		New Zealand	n/a	New Zealand Maori and Pacific populations have 4.2 and 5.6 times the prevalence compared with New Zealand Caucasian population	12.5 (149.5 ± 19.3 months) in boys/ 11 (132.6 ± 16.7 months) in girls
Herngren et al., 2017 [12]	2007–2013	Sweden	9–15	Girls: 44.0 Boys: 57.0 *	12.9 13.0 (3.8–17.7)/11.7 (7.2–15.4)
Beharry et al., 2023 [25]	2008–2018	Caribbean	n/a	2.2	n/a
Perry et al., 2017 [26]	1990–2013	United Kingdom	0–16	4.8	13/12
Longo et al., 2021 [27]	2001–2015	Italy	0–19	2.9	12.55 ± 2.2/ 13 ± 2:11.5 ± 2
Ripatti et al., 2023 [28]	2004–2014	Finland	0–19	Girls: 1.06 Boys: 1.35	n/a
Bouchard et al., 2025 [29]	2024	North America, Europe, Asia, and Oceania	<18	9.62	12.0

Values are reported as in the original publications. NR—not reported; NA—not applicable. Incidence is expressed per 100,000. Age bands and sex-specific estimates are shown when available. * Expressed per 100,000 children.

## Data Availability

The data presented in this study are available on reasonable request from the corresponding author. The data are not publicly available due to privacy and ethical restrictions.
